# Post-extrasystolic variation of ST segment and T wave as a mortality risk predictor after myocardial infarction

**DOI:** 10.3389/fphys.2024.1505242

**Published:** 2025-01-17

**Authors:** Ralf J. Dirschinger, Alexander Müller, Petra Barthel, Alexander Steger, Michael Dommasch, Axel Bauer, Karl-Ludwig Laugwitz, Georg Schmidt, Daniel Sinnecker

**Affiliations:** ^1^ Department of Internal Medicine I, University Hospital rechts der Isar, TUM School of Medicine and Health, Technical University of Munich, Munich, Germany; ^2^ Gefäßpraxis im Tal, Munich, Germany; ^3^ Emergency Department, University Hospital rechts der Isar, TUM School of Medicine and Health, Technical University of Munich, Munich, Germany; ^4^ Clinical Division of Cardiology and Angiology, Innsbruck Medical University, Innsbruck, Tyrol, Austria; ^5^ MVZ Harz, Goslar, Germany; ^6^ TUM School of Medicine and Health, Technical University of Munich, Munich, Germany

**Keywords:** myocardial infarction, ECG analysis algorithm, risk prediction, repolarisation, pest, VPC

## Abstract

**Aims:**

Efficient use of preventive cardiac therapies is often limited by inefficient risk prediction, calling for new prediction tools. Ventricular premature complexes (VPCs) elicit electrocardiographic changes in the repolarization of the first post-extrasystolic normal beat. The aim of this study was to assess whether this *post-extrasystolic ST segment and T wave variation (PEST)* conveys prognostic information regarding the mortality risk of cardiac patients.

**Methods:**

PEST was calculated from 30-min ECGs obtained from 941 survivors of acute myocardial infarction (AMI) as mean difference between the sum of squared voltages from three orthogonal leads (XYZ) of the first (post-extrasystolic) and second (reference) beat after each VPC, in a time window between the limits ϕ_1_ and ϕ_2_. Optimal limits yielding a maximum area under the receiver-operating characteristics (ROC) curve were determined by systematic testing, covering the time window from the J point to the end of the T wave. A strong association was found with ϕ_1_/ϕ_2_ encompassing 40–230 ms after the J point, which was used to calculate PEST in the analysis. Kaplan-Meier curves and univariable/multivariable Cox proportional hazards models were used to study mortality prediction by PEST. The findings were validated in an independent cohort of 1.788 general population subjects aged 60 years or older.

**Results:**

The area under the ROC curve for PEST was 0.72, with an optimum cutoff at ≤ −6.69 mV^2^. The 88 patients with PEST values below this cutoff had a considerably higher mortality than the remainder of the patients (25% vs. 5.8%, p < 0.0001; univariable hazard ratio 4.7, 95% CI 2.4–12.0, p < 0.001). In a multivariable Cox regression analysis considering left-ventricular ejection fraction, presence of diabetes mellitus, and Global Registry of Acute Coronary Events (GRACE) score, PEST remained significantly associated with mortality (hazard ratio 3.6, 95% CI 1.9–6.9, p < 0.0001). In the validation cohort, abnormal PEST was also associated with significantly increased 4-year mortality (11.9% vs. 4.3%, p = 0.00095).

**Conclusion:**

PEST is a strong independent predictor of all-cause mortality in AMI survivors and elderly subjects from the general population. While the pathophysiology of this association remains to be investigated, PEST may complement current risk prediction tools in various clinical settings.

## 1 Introduction

Patients who survive myocardial infarction (MI) retain a substantial risk of mortality due to various conditions such as re-infarction, heart failure, arrhythmia or other MI-related co-morbidities (Fox et al., 2010). It is widely acknowledged that risk stratification is of utmost importance in this patient group to guide effective and economic preventive measures, and that current risk stratification strategies are not optimal (Dagres and Hindricks, 2013).

It has been recognized early that frequent ventricular premature contractions (VPCs) signify increased long-term mortality risk in survivors of acute myocardial infarction (The Coronary Drug Project Research Group, 1973; Kotler et al., 1973; Ruberman et al., 1977). However, while the absence of VPCs in Holter recordings is generally a sign of good prognosis, the presence of VPCs has limited specificity and confers only moderately-increased relative risk (Moss, 1997).

Myocardial infarction disrupts normal autonomic control of heart rhythm and is characterized by reduced parasympathetic tone, sympathetic overactivation and remodeling of the cardiac autonomic innervation (Goldberger et al., 2019). Previous work from our group suggests that the mere presence of VPCs does not necessarily confer increased cardiovascular risk, but that the perturbations of sinus rhythm by VPCs may unmask pathological autonomous patterns, which can be used for risk-prediction. This is exemplified by the discovery of the strong risk predictor *heart rate turbulence (HRT,* Schmidt et al., 1999) with *turbulence onset (TO)* and *turbulence slope (TS)* (Bauer et al., 2008), as well as *post-extrasystolic blood pressure potentiation (PESP)* (Sinnecker et al., 2013).

Very interesting in this regard is the finding of cardiology pioneer Paul Dudley White more than a hundred years ago that VPCs elicit slight electrocardiographic changes in the T wave of the first normal beat after the VPC (White, 1915). The aim of this study was to assess whether this post-extrasystolic T wave change or, more precisely, *post-extrasystolic ST segment and T wave variation (PEST)* may also convey prognostic information. To this end, we systematically investigated the potential association between PEST and all-cause mortality in a cohort of 941 survivors of acute MI and identified that the average voltage deviation within a distinct time window in the ST segment and T wave was a strong and independent mortality predictor. The predictive ability of this PEST parameter could be reproduced in a validation cohort of 1.788 elderly subjects from the general population, indicating that PEST is a promising new tool for the prediction of mortality risk in a broad spectrum of patients.

## 2 Methods

### 2.1 Study cohorts

This work is based on data from two prospective cohort studies. Data from the ART (*Autonomic Regulation Trial*) study of survivors of myocardial infarction (Barthel et al., 2013) were used as the derivation cohort to develop the method for PEST quantification and to assess the prognostic value of PEST in conjunction with other risk predictors in post-infarction patients. To validate the results in an independent cohort, data from the INVADE (*Intervention Project on Cerebrovascular Disease and Dementia in the District of Ebersberg*) study of general population subjects aged 60 years or older (Steger et al., 2020) were used as the validation cohort. All study participants provided written informed consent for participation in the studies. Both studies complied with the Declaration of Helsinki and were approved by the local ethics committees.

### 2.2 Derivation cohort

The ART study (registered as NCT00196274 at ClinicalTrials.gov) has been described previously (Barthel et al., 2013; Sinnecker et al., 2014a). Briefly, a total of 941 consecutive survivors of the acute phase of myocardial infarction aged ≤80 years who were in sinus rhythm and not eligible for implantable cardioverter-defibrillator (ICD) for secondary prevention before hospital discharge were recruited in two Hospitals in Germany (Klinikum rechts der Isar and Deutsches Herzzentrum München) between May 2000 and March 2005 and followed for a median of 6.3 years. The primary endpoint was all-cause mortality. Before hospital discharge, the patients underwent a 30-min recording of a high-resolution ECG (1.6-kHz sampling of orthogonal XYZ leads in Frank’s configuration; TMS International, Enschede, Netherlands). These 30-min ECGs were used to investigate PEST as a predictor of mortality risk.

### 2.3 Validation cohort

For the population-based INVADE study, which was also reported previously (Steger et al., 2020), a total of 1956 subjects from the German district of Ebersberg in Bavaria, insured by Bavaria’s largest health insurance *Allgemeine Ortskrankenkasse (AOK) Bayern*, aged ≥60 years, were recruited between August 2013 and February 2015 and followed up over a median period of 4.0 years (interquartile range 3.6–4.3 years). The primary endpoint was all-cause mortality. After enrollment, all subjects underwent a 30-min ECG recording (5-electrode ECGs with leads I, II, III, aVR, aVL, aVF, V1, sampled at 300 Hz) at their General Practitioner’s office, which was used to calculate PEST. We limited our analysis to the 1788 patients who had sinus rhythm throughout the 30-min ECG recording.

### 2.4 PEST determination

**FIGURE 1:**
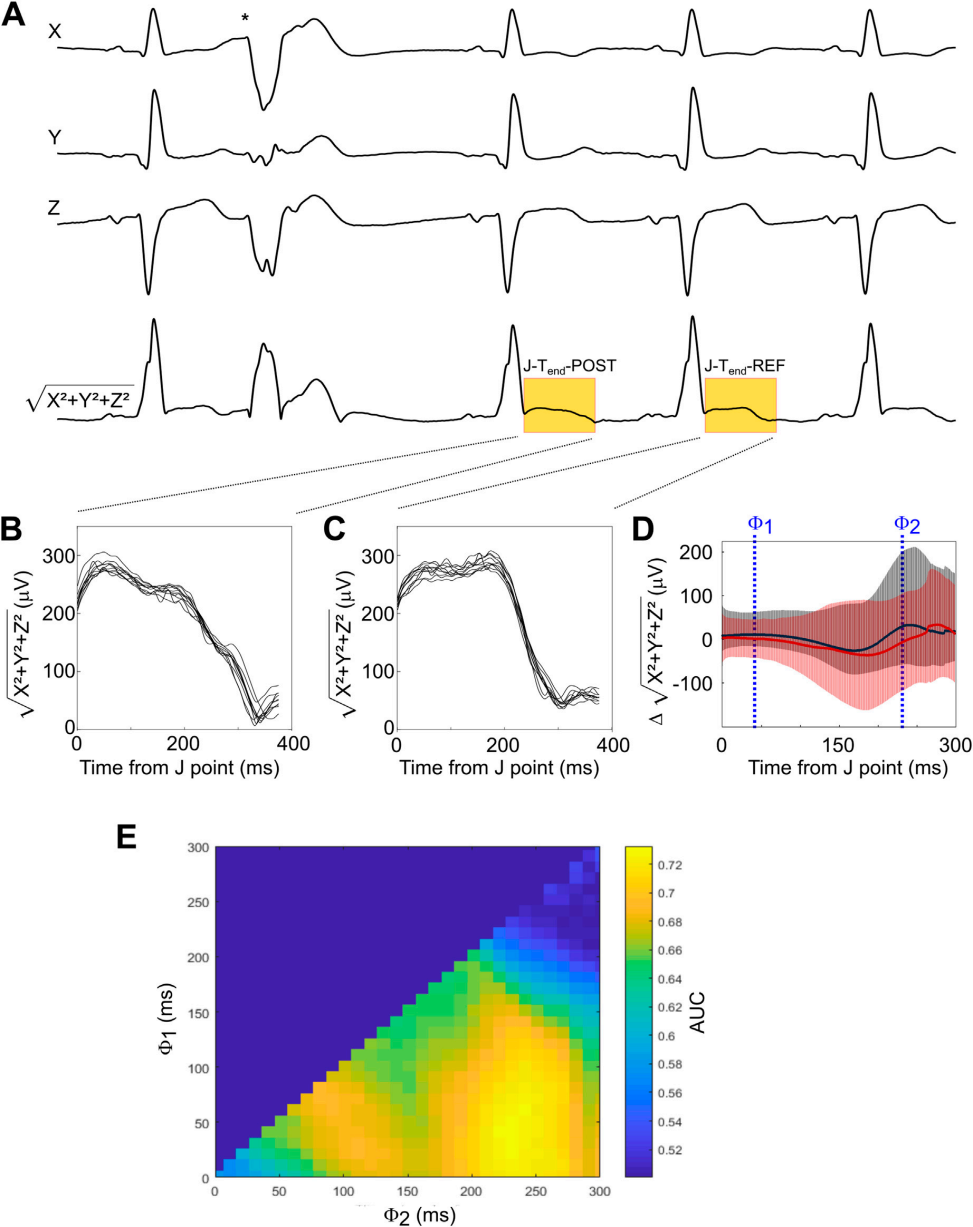
Development of the method for PEST quantification **(A)** A representative ECG recording from the three leads X, Y, and Z showing a typical sequence surrounding a VPC (indicated by asterisk) is depicted, together with the squared sum signal (X^2^+Y^2^+Z^2^) from which PEST was derived. In this signal, the segments from the J point to T_end_ of the first post-extrasystolic beat (termed J-T_end_-POST) and of the reference beat (termed J-T_end_-REF) are highlighted in yellow. **(B, C)** All J-T_end_-POST **(B)** and J-T_end_-REF **(C)** segments from the same patient are shown. **(D)** The difference signal (J-T_end_-POST - J-T_end_-REF) is shown for all patients of the ART cohort. The red line shows the average of patients who died during follow-up, while the average of the survivors is depicted in black. Error bars represent 95% confidence intervals. Examples of threshold values ϕ_1_ and ϕ_2_, between which the difference signal was integrated to obtain a scalar PEST value, are indicated. **(E)** Optimization landscape generated by systematically testing all possible ϕ_1_/ϕ_2_combinations (with ϕ_2_ > ϕ_1_) in 10 ms steps. The area under the receiver-operating characteristics (ROC) curve (AUC) for the prediction of 5-year all-cause mortality by scalar PEST calculated using the respective ϕ_1_/ϕ_2_combinations is shown as a colour-coded heat map.

All ECGs underwent a two-step analysis consisting of 1) an automated analysis to classify each heartbeat as normal or atrial or ventricular premature complexes as well as annotate the J point (the transition between the QRS complex and the ST segment) and the end of the T wave (T_end_), followed by 2) manual review by experienced technicians to eliminate artifacts and correct annotations where necessary. For the quantification of PEST, we compared the ST segment and T wave of the first sinus complex after the VPC to the ST segment and T wave of the second sinus complex after the VPC, serving as reference. The use of other normal sinus complexes as reference beats yielded similar results (not shown). In each recording, all sequences of a single VPC followed by two normal sinus complexes undisturbed by artifacts were identified. From the three leads X, Y, and Z, a single combined signal was generated by calculating the square root of the sum of the squared voltages from the single leads √(X^2^+Y^2^+Z^2^), thereby eliminating negative values. An example of ECG tracings and the square root of the squared sum signal showing a VPC sequence is shown in Figure 1A. The segment between the J point and the end of the T waves of the first and second normal beat after the VPC were compared (J-T_end_-POST, Figure 1B and J-T_end_-REF; Figure 1C, respectively). PEST was calculated as the difference J-T_end_-POST–J-_Tend_-REF. The resulting difference signal, averaged over all VPC sequences, is shown for all patients of the ART cohort in Figure 1D, with signals from patients who were still alive after 5 years of follow up depicted in black, and signals from patients who died during follow up depicted in red. This difference signal can be regarded as a function of time measured from the J point, i.e., it is a one-dimensional matrix quantifying the squared voltage difference between the post-extrasystolic and reference beat at different time points between J and T_end_. In order to derive a scalar PEST value from this matrix, we integrated the matrix between two limits ϕ_1_ and ϕ_2_ by calculating the mean value during this interval (see Figure 1D). Assuming that predictive information present within the J-T_end_ interval may not be temporally equally distributed, we empirically determined how variation of ϕ_1_ and ϕ_2_ affected the prediction of all-cause mortality. This was done by systematically testing all combinations of ϕ_1_ and ϕ_2_ (with ϕ_1 _< ϕ_2_) in 10-ms-increments by calculating scalar PEST for each sequence, averaging the scalar PEST values from different sequences for each patient, and calculating the area under the receiver-operating characteristics (ROC) curve (AUC) for the prediction of all-cause mortality in the whole cohort by PEST calculated using the given combination of ϕ_1_ and ϕ_2_ values. A two-dimensional colour-coded heatmap showing the AUC values achieved with all tested ϕ_1_/ϕ_2_ combinations is shown in Figure 1E. Based on this heatmap, we chose to use the combination of ϕ_1_ and ϕ_2_ of 40 and 230 ms (i.e., to integrate the T wave change in the segment starting 40 ms after the J point and ending 230 ms after the J point) to calculate the scalar PEST values to be used in the remainder of the study. Consequently, for the remainder of the manuscript, the term “PEST” is used for the scalar PEST value calculated with ϕ_1_/ϕ_2_ at 40/230 ms.

### 2.5 Other risk predictors

The Global Registry of Acute Coronary Events (GRACE) score for the prediction of 6-month post-discharge mortality was calculated as described previously (Eagle et al., 2004) and dichotomized at 120 points (Sinnecker et al., 2014b). Periodic repolarization dynamics was assessed using the wavelet method (PRD_wavelet_) as described previously (Rizas et al., 2014) and dichotomized at ≥5.72 deg^2^. QT duration corrected for heart rate (QTc) was calculated by Bazett’s Formula (Bazett, 1920) and dichotomized at ≥450 ms.

### 2.6 Validation

To validate the prognostic potential of PEST in the INVADE cohort, we applied the above-described algorithm without any changes or further optimization to ECGs from the INVADE cohort. To ensure compatibility of the algorithm, the ECG was up-sampled to 1.6 kHz, and instead of the XYZ leads which were not available in this cohort, we used the ECG leads III, aVR and V1 for the subsequent analysis. We present Kaplan-Meier estimates of survival for PEST dichotomized at the optimum cutoff determined in the ART study.

### 2.7 Statistics

The distribution of quantitative data is presented by median and inter-quartile range (IQR). Qualitative data is described by absolute and relative frequencies. Based on the ROC curve, the optimum cutoff for PEST was chosen by maximizing Youden’s index J = Sensitivity + Specificity-1 (Youden, 1950). Survival curves are presented as Kaplan-Meier estimates. The statistical significance of differences between group-specific Kaplan-Meier survival estimates was assessed by Log-rank tests. Univariable and multivariable Cox proportional hazards models were used to calculate hazard ratios (with 95% confidence intervals) for predictors of all-cause mortality. R 3.0.1 (R foundation for Statistical Computing, Vienna, Austria) was used for all statistical analyses. Differences were considered statistically significant if *p* < 0.05.

## 3 Results

### 3.1 Derivation cohort

The ART cohort, which has been previously described (Sinnecker et al., 2014a), consisted of 941 survivors of acute myocardial infarction (19.3% females) who predominantly received percutaneous coronary intervention (93.3%; Table 1). The vast majority of patients was treated with Aspirin (97%), Clopidogrel (97.8%), beta blockers (95.3%), ACE inhibitors (94.0%) and CSE inhibitors (93.4%). In the 30-min ECG recordings of 242 patients (25.7%), VPCs with undisturbed post-ectopic ECG segments were present that allowed the assessment of PEST (as defined in the methods section). These patients (termed *“PEST calculable”*) were significantly older (median age 64.9 vs. 59.5 years) and had a significantly lower left-ventricular ejection fraction (49% vs. 54%) than the remaining patients in whom PEST could not be calculated, predominantly because of absent or rare VPCs (termed *“PEST incalculable”*, see Table 1). The median VPC frequencies in the *“PEST calculable”* and *“PEST incalculable”* groups were eight per hour and 0 per hour, respectively (see Table 1).

**TABLE 1:** Baseline characteristics.

	All patients (n = 941)	PEST calculable (n = 242)	PEST not calculable (n = 699)	P (PEST calculable vs. PEST not calculable)	PEST normal (n = 154)	PEST abnormal (n = 88)	P (PEST normal vs. PEST abnormal)
Age (years, median [IQR])	60.9 [51.6–68.8]	64.9 [57.2–71.3]	59.5 [50.2–67.6]	<0.001	64.6 [57.0–70.5]	65.8 [58.8–72.7]	0.475
Females, n (%)	182 (19.3)	41 (16.9)	141 (20.2)	0.316	28 (18.2)	13 (14.8)	0.616
GRACE score (points, median [IQR])	110.2 [93.4–125.8]	118.6 [102.6–134.7]	107.2 [90.3–123.7]	<0.001	118.0 [101.2–134.7]	119.1 [104.5–133.7]	0.810
LVEF (percent, median [IQR])	53 [45–60]	49 [40–57.75)]	54 [46–61]	<0.001	49 [40–57]	50 [37.75–59.25]	0.788
Diabetes mellitus, n (%)	184 (19.6)	49 (20.2]	135 (19.3)	0.824	34 (22.1)	15 (17.0)	0.441
Creatinine (mg/dL, median [IQR])	1.1 [0.9–1.3]	1.1 [1.0–1.3]	1.1 [0.9–1.3]	0.124	1.1 [1.0–1.3]	1.2 [1.0–1.3]	0.358
Acute Intervention, n (%)							
PCI	878 (93.3)	223 (92.1)	655 (93.7)	0.439	140 (90.9)	83 (94.3)	0.484
CABG	6 (0.6)	2 (0.8)	4 (0.6)	1.0	2 (1.3)	0 (0.0)	0.737
Thrombolysis	14 (1.5)	2 (0.8)	12 (17)	0.498	2 (1.3)	0 (0.0)	0.737
None	43 (4.6)	15 (6.2)	28 (4.0)	0.219	10 (6.5)	5 (5.7)	1.0
CK_max_ (U/L, median [IQR])	1,302 [646–2,460]	1,285 [672–2,540]	1,315 [637–2,445]	0.861	1,440 [804–2,578]	1,101 [488.5–2,180]	0.058
Aspirin, n (%)	913 (97.0)	235 (97.1)	685 (96.7)	0.455	151 (98.1)	86 (97.7)	1.0
Clopidogrel, n (%)	920 (97.8)	235 (97.1)	685 (98.0)	0.579	150 (97.4)	85 (96.6)	1.0
Beta blocker, n (%)	897 (95.3)	233 (96.3)	664 (95.0)	0.521	151 (98.1)	82 (93.2)	0.484
ACE inhibitor, n (%)	885 (94.0)	235 (97.1)	650 (93.0)	0.030	150 (97.4)	85 (96.6)	1.0
CSE inhibitor, n (%)	897 (93.4)	227 (97.9)	652 (93.3)	0.894	144 (93.5)	83 (94.3)	1.0
VPC per hour (median [IQR])	0 [0–2]	8 [2–23.5]	0 [0–0]	<0.001	6 [2–19.5]	12 [4–36]	0.021

PEST, post-extrasystolic ST segment/T wave variation; IQR, inter-quartile range; GRACE, global registry of acute coronary events; LVEF, left-ventricular ejection fraction; PCI, percutaneous coronary intervention; CABG, coronary artery bypass grafting; CK, creatine kinase; VPC, ventricular premature complex.

### 3.2 Risk prediction in post-infarction patients by PEST

**FIGURE 2:**
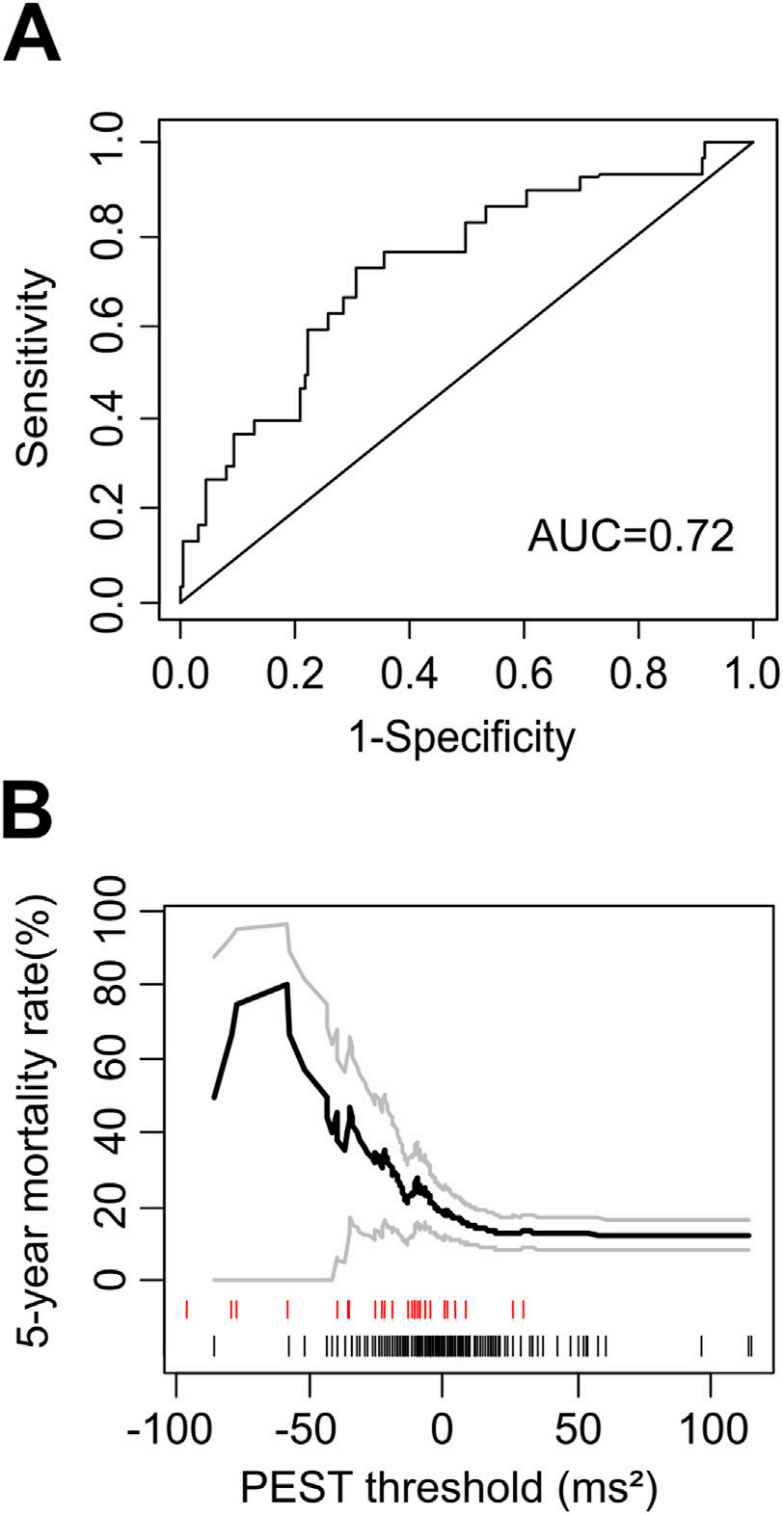
Mortality risk prediction by PEST as continuous variable. **(A)** Receiver-operating characteristic (ROC) curve for the prediction of 5-year all-cause mortality in the ART cohort by PEST. The area under the ROC curve (AUC) is indicated. **(B)** Continuous association of PEST with mortality. The 5-year mortality rate estimated by the Kaplan-Meier method in patients with PEST less than or equal to a certain threshold is plotted as a function of this threshold. Grey curves show 95% confidence limits of the mortality estimate. The marks below the graph indicate PEST values measured in single study participants: the black marks correspond to patients who were still alive at the end of follow-up, while the red marks correspond to patients who died during follow-up.

The area under the ROC curve for the prediction of all-cause mortality by PEST was 0.72, indicating a strong association between PEST and mortality **(** Figure 2A). The distribution of PEST values in survivors and non-survivors is shown in Figure 2B. Both positive and negative PEST values were observed, i.e., the mean voltage in the specified time window was either larger or smaller in the first post-ectopic normal heartbeat compared to the reference beat. On average, PEST values of non-survivors were shifted towards the negative compared to survivors, resulting in a continuous association of PEST values with mortality, with more negative values being associated with an increased mortality rate (see Figure 2B). The optimum cutoff based on Youden’s Index J (Youden, 1950) was ≤ −6.69 mV^2^. Of the 242 patients in whom PEST was calculable, 88 patients (36.4%) had PEST values smaller than or equal to −6.69 mV^2^ (termed *“PEST abnormal”* in the following text), and 154 (63,3%) had values greater than −6.69 mV^2^ (termed *“PEST normal”*). The clinical characteristics of patients with normal and abnormal PEST are shown in Table 1.

**FIGURE 3:**
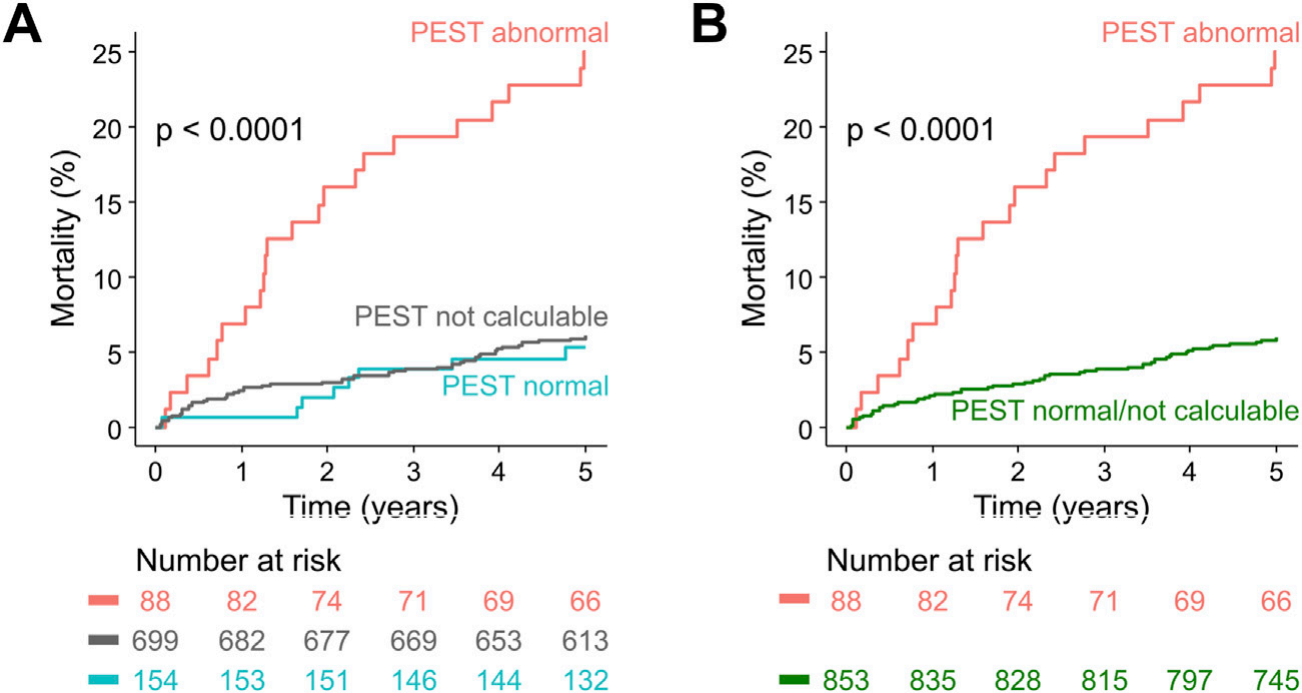
Survival in post-infarction patients stratified according to PEST. **(A)** Kaplan-Meier curves are shown for patients from the ART cohort with abnormal PEST, patients in whom PEST was not calculable, and patients with normal PEST. The number of patients at risk in the respective groups are shown below the graph in the same colour-coding. **(B)** Same as in **(A)**, but the groups with normal PEST and with not calculable PEST were combined.

Considering only patients in whom PEST was calculable, abnormal PEST was associated with a 5-year mortality rate of 25% (22/88), compared to 5.2% (8/154) in patients with normal PEST (p < 0.0001; Figure 3A). In univariable Cox regression, abnormal PEST was a highly-significant predictor of 5-year all-cause mortality (hazard ratio 5.3, 95% CI 2.4–12.0, p < 0.001).

Patients in whom PEST was incalculable (which was predominantly due to absence or scarcity of VPCs in the 30-min ECG) had a 5-year mortality rate of 6.0% (42/699), comparable to those with normal PEST (see Figure 3A). Therefore, as previously applied for VPC-related predictors (Bauer et al., 2008; Sinnecker et al., 2014b), the groups with normal PEST and with incalculable PEST were combined for all subsequent analyses, allowing to assess the prognostic potential of abnormal PEST in the whole study cohort (Figure 3B). In univariable Cox regression performed on the whole cohort of 941 patients, abnormal PEST was associated with a hazard ratio of 4.7 (95% CI 2.4–12.0, p < 0.001). The results of univariable and multivariable Cox regression analysis considering also the well-established risk predictors left-ventricular ejection fraction, presence of Diabetes mellitus, and the Global Registry of Acute Coronary Events (GRACE) score (Eagle et al., 2004) are shown in Table 2. These results show that PEST conveys independent prognostic information that is complementary to that provided by the established risk factors.

**TABLE 2:** Univariable and Multivariable Cox Regression: PEST and clinical risk predictors.

Variable	Univariable analysis		Multivariable analysis	
	Hazard ratio (95% CI)	p-value	Hazard ratio (95% CI)	p-value
PEST abnormal	4.7 (2.9–7.8)	<0.0001	3.6 (1.9–6.9)	<0.0001
LVEF ≤35%	4.0 (2.3–6.9)	<0.0001	2.5 (1.4–4.4)	0.0019
VPC count ≥10 per hour	2.9 (1.8–4.9)	<0.0001	0.88 (0.45–1.7)	0.71
GRACE score ≥120 points	5.8 (3.4–9.9)	<0.0001	4.8 (2.8–8.4)	<0.0001

CI, confidence interval; PEST, post-extrasystolic ST segment/T wave variation; LVEF, left-ventricular ejection fraction; VPC, ventricular premature complex; GRACE, global registry of acute coronary events.

### 3.3 Relation of PEST to other repolarization-related risk predictors

We also assessed whether PEST contained additional prognostic information compared to the established ECG-based repolarization-related risk predictors periodic repolarization dynamics (PRD) and heart rate-corrected QT duration (QTc) using multivariable Cox analysis. As shown in Table 3, PEST remained a highly-significant mortality risk predictor in multivariable analyses considering PRD and QTc, with a multivariable hazard ratio of abnormal PEST of 3.46 (95% CI 2.6–5.81; p < 0.0001), indicating that PEST contains additional predictive information that is not redundant to that provided by PRD or QTc.

**TABLE 3:** Univariable and Multivariable Cox Regression: periodic repolarization dynamics (PRD), frequency-corrected QT duration (QTc) and PEST.

Variable	Univariable analysis		Multivariable analysis	
	Hazard ratio (95% CI)	p-value	Hazard ratio (95% CI)	p-value
Continuous variables				
PEST (per mV^2^)	0.97 (0.96–0.98)	<0.0001	0.97 (0.96–0.98)	<0.0001
PRD_wavelet_ (per deg^2^)	1.14 (1.10–1.19)	<0.0001	1.16 (1.09–1.24)	<0.0001
QTc (per ms)	1.011 (1.005–1.016)	<0.0001	1.004 (0.995–1.013)	0.418
Dichotomized variables				
PEST abnormal	4.71 (2.85–7.78)	<0.0001	3.46 (2.6–5.81)	<0.0001
PRD_wavelet_ ≥ 5.75 deg^2^	4.75 (2.94–7.67)	<0.0001	3.62 (2.21–5.94)	<0.0001
QTc ≥450 ms	2.99 (1.88–4.74)	<0.0001	2.29 (1.42–3.70)	0.0007

PEST, post-extrasystolic ST, segment/T wave variation; CI, confidence interval; PRD_wavelet_, periodic repolarization dynamics calculated using the wavelet method; QTc, QT, duration corrected for the Heart rate using Bazett’s formula.

### 3.4 Validation

**FIGURE 4:**
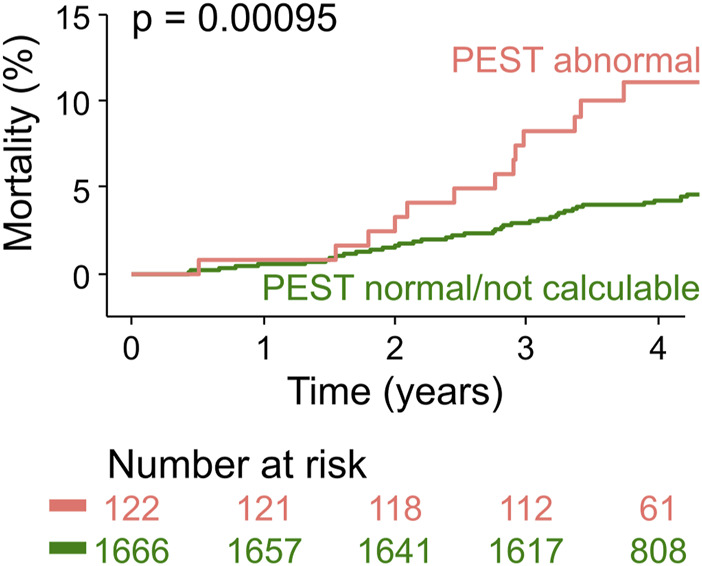
Validation of PEST as a mortality risk predictor in the population-based INVADE cohort. Kaplan-Meier curves are shown for participants of the INVADE cohort stratified according to PEST. The number of patients at risk in the respective groups are shown below the graph in the same colour-coding.

To validate PEST as a mortality risk predictor in an independent cohort, we assessed PEST using the above-described algorithm and cut-point without any further optimization in 30-min ECGs from participants of the population-based INVADE study (Steger et al., 2020). As expected, in this cohort of elderly people from the general population, the proportion of study subjects with abnormal PEST was smaller than in the post-infarction patients of the ART cohort (6.8% vs. 9.4%). During the median follow-up of 4 years, 82/1788 study participants died (4.6%). Survival curves for patients with abnormal PEST and with normal/not calculable PEST are depicted in Figure 4, showing a significantly higher mortality rate in those with abnormal PEST (11.9% vs. 4.3%).

## 4 Discussion

In this study, we demonstrate that the post-extrasystolic variation of ST segment and T wave (PEST) after spontaneous ventricular premature complexes (VPCs) conveys prognostic information that allows risk stratification with respect to all-cause mortality in survivors of acute myocardial infarction and in not preselected elderly individuals from a general population cohort. In post infarction patients, PEST was associated with an almost five-fold increase of mortality compared to patients with normal PEST or absence of VPCs (25% vs. 5%; see Figure 3; Table 2). Moreover, in a population-based validation cohort of elderly subjects, abnormal PEST was still associated with a 2.8-fold increase of 4-year mortality rate (11.9% vs. 4.3%; see Figure 4).

The prognostic information provided by PEST was independent from established risk predictors such as *LVEF, VPC count*, or *GRACE score*, as shown by multivariable Cox regression analysis (see Table 2) and it was independent from established risk-associated repolarisation parameters as *QTc* or *PRD* (see Table 3).

The results of this study may have broad clinical implications. In patients with heart disease such as survivors of acute myocardial infarction, risk stratification is an important aspect of patient care. An area in which risk stratification strategies have direct therapeutic impact is the selection of patients for prophylaxis with implantable cardioverter-defibrillators (ICDs), which may be life-saving in patients at risk of arrhythmic sudden cardiac death. Current selection, which is based mainly on left-ventricular ejection fraction, results in a high number needed to treat (NNT) to prevent arrhythmic death (i.e., most patients who get an ICD for primary prophylaxis based on current criteria will never need it), while most post-infarction patients who are found to die suddenly did not fulfil the implantation criteria before their death. Improvement of the underlying risk prediction is urgently needed.

PEST, as calculated by the method presented in this manuscript, is an easily-applicable parameter that may help improve risk stratification. Our study is not able to reliably address the question whether the predictive quality of PEST was specifically driven by sudden cardiac deaths in the cohorts, as the pathophysiology of other repolarization abnormalities would suggest. Although abnormal PEST was in fact associated with sudden cardiac mortality but not non-sudden cardiac mortality in our study, the number of endpoints was too low to validly draw this conclusion (not shown). If PEST could be proven to predict sudden cardiac death in future studies, risk assessment by PEST could possibly prevent sudden cardiac deaths without increasing the numbers of implanted ICDs. Therefore, the relation of PEST and sudden cardiac death needs to be studied.

The prognostic value of the PEST parameter developed in the ART cohort was validated in an independent cohort of 1788 elderly subjects from the general population (INVADE cohort). This underlines that our PEST parameter is a robust mortality predictor and largely rules out that the strong performance of PEST as a risk predictor was a result of over-optimization in the derivation cohort. Moreover, it shows that PEST is not limited to predicting the mortality of survivors of acute myocardial infarction, but is also capable of identifying elderly subjects from the general population at increased mortality risk. The applicability to further clinical settings, e.g., risk prediction in younger patients with myocarditis or cardiomyopathy, remains to be investigated.

Since the phenomenon of PEST has been first described more than a century ago (White, 1915), numerous attempts have been made to link postextrasystolic repolarization abnormalities to specific clinical settings. For instance, in a case-control study of 41 survivors of ventricular fibrillation or hemodynamically unstable ventricular tachycardia, postextrasystolic U wave changes, but not postextrasystolic T wave changes were identified as independent predictors of ventricular tachyarrhythmias (Viskin et al., 1996). To our knowledge, the present study is the first prospective study showing that post-extrasystolic variation of ST segment and T wave can predict clinical outcomes.

The pathophysiologic basis of the predictive information within PEST remains elusive. Given that repolarization of the cardiomyocytes is an active, energy-consuming process, pathological PEST may well be related to myocardial ischemia. However, prior attempts to link specific patterns of post-extrasystolic T wave changes to coronary artery stenosis were not successful (Engel et al., 1977). Moreover, in the ART cohort, in which all patients underwent coronary angiography after their acute myocardial infarction, PEST values did not significantly differ between patients who were completely revascularized and those who were not at the time of PEST assessment (data not shown).

Based on our data, we cannot judge whether the PEST pattern we identified as being linked to increased mortality risk is due to regional repolarization abnormalities within the myocardium that project into the ECG sum vector, or whether it is the result of a cellular event that is present throughout the ventricular myocardium. Assuming that abnormal PEST possibly indicates the risk of arrhythmic events, it may be a measure of “relative” VPC prematurity in relation to the individual electric vulnerability of the post-ischemic myocardium. In other words, post-extrasystolic ST segment/T wave variation may occur when relevant portions of the myocardium are still refractory at the timing of VPC onset and therefore behave differently throughout the VPC and the post-extrasystolic beat. Alternatively, PEST may be associated with ischemia-induced alterations in cardiac conduction, which relies on distinct heterogeneous subcellular and cellular localisations of gap junctions, ion channels, and autonomic nerves (Qu et al., 2024). Moreover, the phenomenon of PEST bears resemblance to the phenomenon of cardiac memory–T wave changes elicited by alterations in the sequence of ventricular activation, e.g., due to ventricular pacing–which has been linked to alterations in transient outward (*I*_to_) current (Yu et al., 1999). The postextrasystolic pause and subsequent alteration of this current may be a possible explanation for the observed changes. Experimental studies such as animal experiments, experiments on *ex vivo* myocardial preparations, and *in silico* simulation studies may be necessary to gain a deeper understanding of the pathophysiological basis of PEST.

Some post-extrasystolic variations of the ST segment and T wave can be observed by eye and may include slight changes of T amplitude, T vector, ST segment deviation or QT duration, and not all may be visible in the same individuals. However, when analysed as single parameters, the presented PEST parameter showed the best predictive performance (data not shown). Yet, it seems plausible that our proposed parameter actually reflects and summarizes more than one physiologic process.

As most cellular processes underlying the surface ECG are heart rate dependent, it is obvious that the presented method of PEST measurement within a fixed time window after the J point will not perfectly reflect physiology (see *Methods, PEST determination*). Nevertheless, we found that a rate corrected algorithm was more laborious and slightly more prone to error (due to the sometimes-difficult annotation of T_end_) while not improving the predictive performance of PEST (data not shown). This lack of improvement is likely due to the “permissive” borders of the time window of interest: ϕ_1_ and ϕ_2_ values encompassing a time window between 20–70 ms and 220–260 ms after the J point yielded a similar strength of prediction (see Figure 1E). For the ϕ_1_/ϕ_2_ window finally chosen, resting heart rate differences appear to be reflected well enough to retain the predictive information throughout the cohorts. Prioritising reproducibility and clinical feasibility over ideal pathophysiologic representation for this clinical prediction tool, we decided not to use a rate corrected algorithm in our parameter.

### 4.1 Limitations

The current study has limitations. The method developed here represents only one of several possible ways to extract the prognostic information from the post extrasystolic T wave change. Our approach was based on traditional systematical-empirical testing of an observed phenomenon, focused on one particular aspect of PEST, i.e., amplitude. It has to be pointed out that an unbiased machine-learning approach may be able to extract even stronger predictive information. Nevertheless, our method is straightforward, reproducible and readily-applicable to Holter ECGs available in many doctors’ offices, which might be of particular interest in the light of our finding of a successful prediction of mortality in a general population cohort.

In our study, we did not perform serial measurements and therefore cannot judge on the stability or a potential reversibility of abnormal PEST over time.

While the putative pathophysiology of PEST may suggest a possible specificity in the prediction of sudden cardiac death, the number of these endpoints in this cohort was insufficient to prove this claim. However, for an optimal use of PEST in clinical risk prediction, addressing this question in a larger population of cardiac patients will be required.

### 4.2 Conclusion

In summary, the novel PEST parameter presented in this study is a strong and independent predictor of all-cause mortality in survivors of acute myocardial infarction as well as in elderly subjects from the general population. While the pathophysiological mechanism of this association remains to be investigated, PEST might serve as a complement to current risk prediction tools in various clinical settings.

## Data Availability

The data analyzed in this study is subject to the following licenses/restrictions: The original datasets of the studies analyzed for the current study may be obtained via the corresponding authors. Requests to access these datasets should be directed to Georg Schmidt, gschmidt@tum.de.
